# Anti-Glioma Effect of *Pseudosynanceia Melanostigma *Venom on Isolated Mitochondria from Glioblastoma Cells

**DOI:** 10.31557/APJCP.2021.22.7.2295

**Published:** 2021-07

**Authors:** Maral Ramezani, Fatemeh Samiei, Jalal Pourahmad

**Affiliations:** *Department of Toxicology and Pharmacology, School of Pharmacy, Shahid Beheshti University of Medical Sciences, Tehran, Iran. *

**Keywords:** Mitochondria, glioblastoma, fish venoms, cell line- tumor, toxicity, Persian Gulf

## Abstract

**Background:**

Glioblastoma is the most common primary malignant tumor of the central nervous system that occurs in the spinal cord or brain. *Pseudosynanceia Melanostigma *is a venomous stonefish in the Persian Gulf, which our knowledge about is little. This study’s goal is to investigate the toxicity of stonefish crude venom on mitochondria isolated from U87 cells.

**Methods:**

In the first stage, we extracted venom stonefish and then isolated mitochondria have exposed to different concentrations of venom. Finally, mitochondrial toxicity parameters (Succinate dehydrogenase (SDH) activity, Reactive oxygen species (ROS), cytochrome c release, Mitochondrial Membrane Potential (MMP), and mitochondrial swelling) have evaluated.

**Results:**

To determine mitochondrial parameters, we used 115, 230, and 460 µg/ml concentrations. The results of our study show that the venom of stonefish selectively increases upstream parameters of apoptosis such as mitochondrial swelling, cytochrome c release, MMP collapse and ROS.

**Conclusion:**

This study suggests that *Pseudosynanceia Melanostigma *crude venom has selectively caused toxicity by increasing active mitochondrial oxygen radicals. This venom could potentially be a candidate for the treatment of glioblastoma.

## Introduction

Glioblastoma multiform is the most common group of brain tumors and malignant gliomas are the deadliest types of adult brain tumors. Glioblastoma accounts for more than 50% of all types of gliomas. The overall prevalence is 2-3 per 100,000 and 20% of all intracranial tumors, and it is more common in men over 60 years (Wang et al., 2018).

In cellular studies, glioblastoma multiform cells (U87) are very different in shape and size; they have high vascular proliferation and necrosis and increase the involvement of tumor cells in different areas of the brain. This type of tumor has a poor prognosis with various clinical features such as cognitive impairment, personality changes, headache, loss of sensation (paresthesia), and gait imbalance. In the treatment of the disease, this type of cancer is still considered incurable; because despite the common treatments including surgical removal of the tumor and subsequent radiation therapy in addition to drug treatment with Temozolomide (TMZ), people with the disease are usually die within 12 to 15 months (Anjum et al., 2017). One of the treatments for glioma is cytotoxicity as well as induction of apoptosis to inhibit the proliferation of cancer cells (Yan et al., 2012). Therefore, the need for new therapies or drugs seems to be necessary.

Aquatic animals are used for animals that live in either freshwater or saltwater. Marine animals most commonly used for animals live in saltwater, i.e. in oceans, seas, etc. Fish make up about half of the vertebrates on Earth, and there are approximately 44,222 species of fish in 12 orders and 221 families. Among them, there are about 1,422 species of poisonous marine fish, which include biting superficial fish, scorpionfish, zebrafish, stonefish, toadfish, fish astronomers, and species of sharks, mice, fish surgeons (Smith and Wheeler, 2006). Because the venom of various animals, especially fish, has a wide range of pharmacological effects on the human nerves, muscles, and cardiovascular system, venom proteins are a source for the emergence of drugs for the treatment of pain, cancer, infectious diseases, and autoimmune diseases (Smith and Wheeler, 2006). Apoptosis is programmed cell death in which damaged and redundant cells are removed by various mechanisms, thus preventing damage to adjacent healthy cells (McConkey, 1998). Two pathways exist for apoptosis. The mitochondrial pathway of apoptosis begins with any metabolic stress in the cells; the main result is a change in the permeability of the outer membrane of the mitochondria that leads to the exit of cytochrome c. An apoptosome formed in the cytosol and eventually leads to the activation of the caspase cascade (Wang, 2001). Various compounds of marine animals known to use in the treatment of cancer using apoptosis such as Dolastatin 10, Didemnin B and Aplidine, Bryostatin 1, Ecteinascidin-743 (ET-743) and etc.(Beesoo et al., 2014). 

Stonefish (Pseudosynanceia melanostigma) a native of the Persian Gulf that considers as one of the fish that have poison. This fish is about 2-3 cm long and has 2-5 dorsal thorns, 2 thoracics, and 4-6 anal thorns that are equipped with poison glands. This fish lives in muddy, quiet, and shallow coastal areas (Vazirizadeh et al., 2014). This fish’s toxin is a myotoxin that effects on skeletal, cardiac, and involuntary muscles and blocks conduction in these tissues. It also causes the release of acetylcholine, substance P ,and cyclooxygenase (Gwee et al., 1994; Carrijo et al., 2005). Three major venom types have been isolated from different species of stonefish, Stonustoxin (SNTX), verrucotoxin (VTX), and trachynilysin (TLY) (Vazirizadeh et al., 2014). The most important enzymes in the venom include Hyaluronidase, Phospholipase, Acetylcholinesterase and Metalloproteinase enzymes (Austin et al., 1965; Birkedal-Hansen et al., 1993; Kreil, 1995; Dori et al., 2007). The venom can generate severe pain, respiratory arrest, cardiovascular injury, muscle paralysis and convulsions, and even death (Diaz, 2015). Multiple studies have revealed the medicinal properties of each subunit of stonefish’s venom (Carlson et al., 1971; Church and Hodgson, 2000). In a study, researchers represented that this venom has catalytic effects against numerous cell types (Kreger, 1991). In addition, anticancer effects of venom are described by using in Vivo cancer model. They represented that the venom, induced apoptosis in Ehrlich’s associate’s carcinoma (EAC), splenocytes, and P388 cells (Rajeshkumar et al., 2015).

This study goal is to investigate the toxicity of *Pseudosynanceia melanostigma *stonefish crude venom on mitochondria isolated from U87 cells and a comparison of its effect on mitochondria of normal cells.

## Materials and Methods

In this study after determining IC_50_ by applied 0, 100, 200, 500, and 1000 µg/ml crude venom on mitochondria from U87 cells, we had six groups:

1) Control normal mitochondria (mitochondria from normal rat’s brain cells).

2) Control cancerous mitochondria (mitochondria from U87 cells).

3) Normal mitochondria + 230 µg/ml crude venom.

4) Cancerous mitochondria + 115 µg/ml crude venom.

5) Cancerous mitochondria + 230 µg/ml crude venom.

6) Cancerous mitochondria + 460 µg/ml crude venom.

*Chemical and kits*

Dimethyl sulfoxide (DMSO), MTT (3-[4, 5-dimethylthiazol-2-yl]-2, 5-diphenyltetrazolium bromide), 2’,7’-dichlorofluorescein Diacetate (DCFH-DA), rhodamine 123 (RH123), TRIS-HCl, sodium succinate, sucrose, KCl, Na_2_HPO_4_, MgCl_2_, K_2_HPO_4_, EDTA (ethylene diamine tetra acetic acid), EGTA (ethylene glycol-bs (2-aminoethyl ether)-N,N,N’,N’-tetra acetic acid), RPMI 1640, fetal bovine serum (FBS) were purchased from Sigma Aldrich (Taufkrichen, Germany). Quantikine Rat/Mouse Cytochrome c Immunoassay kit was purchased from R & D Systems, Inc. (Minneapolis, Minn.).

*Crude Venom Preparation*

To extract *Pseudosynanceia melanostigma *stonefish venom, stonefish has been caught from the coastal areas of Bushehr beaches. Then, the thorns attached to the venomous glands have cut and incubated for 24 hours in 0.15 M sodium chloride solution at 4°C. After 24 hours, the obtained solution has been centrifuged at 12,000 g for 20 minutes at 4°C and then the supernatant has been collected (Vazirizadeh et al., 2014). The crude venom has been immediately freeze-dried and kept at -20°C until use for tests (Bradford, 1976).

*Isolation of mitochondria from U87 cells*

U87 cells have been purchased from Pasteur Institute (Iran) and mitochondria have been separated by using multiple centrifugation methods. Briefly, after cells have washed with cold PBS, they have centrifuged at 450g. The sediment has been suspended in isolation buffer (70 mM sucrose, 20 mM HEPES, 220 mM D-mannitol, and 1 mM EDTA, pH = 7.2), and then the cells have been lysed by homogenization. Then, cells have been centrifuged at 750g for 20 min. Supernatant has re-centrifuged at 8000g for 15 min. The sediment has been called as mitochondrial fraction and maintained in buffer solution (140 mM KCl, 10 mM NaCl, 2.0 mM MgCl_2_, 0.5 mM KH_2_PO_4_, 20 mM HEPES ,and 0.5 mM EGTA, pH=7.2) (Salimi et al., 2017).

*Isolation of mitochondria from normal rat brain*

After anesthesia, the Male Wistar rat’s (250-300 g) brain has quickly excised and used for the separation of mitochondria. Brain mitochondria have been prepared from rats using the differential centrifugation method. At first, the brains have been removed and torn to pieces in cold mannitol solution (mitochondria isolation buffer) and then have mildly homogenized in a glass handheld homogenizer. Then, the homogenized brain has centrifuged at 1,500 g for 10 min at 4°C. The supernatant has re-centrifuged at 12,000 g for 10 min at 4°C. The mitochondrial sediment has been rinsed by mild suspension in the buffer solution (0.25 M sucrose, 20 mM KCl, 2.0 mM MgCl_2_, 1.0 mM Na_2_HPO_4_, 0.25 M sucrose and 0.05 mM TRIS-HCl, pH = 7.2) at 4°C and centrifuged again at 12,000 × g for 10 min. Finally, mitochondrial sediment has been suspended in Tris buffer (0.05 M Tris-HCl, 0.25 M sucrose, 20 mM KCl, 2.0 mM MgCl_2_, and 1.0 mM Na2HPO4, pH of 7.4) at 4° C and kept for use in mitochondria toxicity tests (Atabaki et al., 2020).

*Determination of Succinate Dehydrogenase (SDH) Activity*

The activity of the mitochondrial succinate dehydrogenase enzyme has measured by the MTT test. In summary, 100 ml of Mitochondrial suspension have been exposed with different concentrations of crude venom (0 - 1,000 µg/ml) for 1 hour at 37°C; then, 50 ml of 0.5% of MTT have been added to the suspension and wrap plate in foil and incubated for 30 minutes at 37°C. Finally, 100 mL of DMSO have been added and then the absorbance at 570 nm measured with an ELISA reader (Hosseini et al., 2013). 

*Measurement of ROS Formation*

The Reactive oxygen species (ROS) formation in mitochondria has performed using the DCFH-DA fluorescent dye. Isolated mitochondria were suspended in respiration buffer (0.32 mM sucrose, 10 mM Tris, 20 mM Mops, 50 M EGTA, 0.5 mM MgCl_2_, 0.1 mM KH_2_PO_4_, 5mM sodium succinate) and then, DCFHDA was added at a final concentration of 10 µM after exposure mitochondrial samples with various concentration of crude venom for 60 minutes and incubation at 37°C. The fluorescence intensity of DCF was assayed at the excitation wavelength of 485 nm and an emission wavelength of 520 nm using fluorescence spectrophotometer (Gao et al., 2009).

*Determination of Mitochondrial Swelling*

Mitochondrial swelling in mitochondrial suspension (1mg protein/ML) carried out by measurement of decreased absorbance of mitochondrial particles by spectrophotometer at 540 nm (Rotem, 2005). Briefly, isolated mitochondria have been suspended in swelling buffer including 70 mM sucrose, 230 mM mannitol, 3 mM HEPES, 2 mM Tris-phosphates, 5mM succinate, and 1M rotenone and incubated at 30°C for 1 hour with different concentration of venom. The absorbance has measured at 540 nm within 1 hour, at 15 minutes time intervals with an ELISA reader. An increase in mitochondrial swelling is characterized by a decrease in mitochondrial absorbance at 540 nm (Talari et al., 2014).

*Determination of Mitochondrial Membrane Potential (MMP)*

MMP collapse was determined using the fluorescent cationic dye Rhodamine 123. After incubating with different concentrations of venom, Rhodamine 123 (final concentration 10 mM) has been added to the suspended mitochondria in MMP assay buffer (220 mM sucrose, 68 mM D-mannitol, 10mMKcl, 5mMKH_2_PO_4_, 2mMMgCl_2_, 50 M EGTA, 5mM sodium succinate, 10 mM HEPES, 2 M Rotenone). The fluorescence activity was determined at the excitation and emission wavelength of 490 nm and 535 nm, respectively (Pourahmad et al., 2010).

*Measurement of Cytochrome C release*

The release of cytochrome c by crude venom has been accomplished according to instructions of the Quantizing Rat/Mouse Cytochrome C Immunoassay kit provided by R and D Systems, Inc. At first, a monoclonal antibody specific from rat/mouse cytochrome c pre-coated into the micro-plate. In addition, 75 μl of conjugate and 50 μl of standard and positive control were added to each well of the micro-plate. According to the kit instructions, we added the 1 μg of protein from each supernatant fraction to the sample wells. All of the standards, controls, and samples were added to two wells of the micro-plate. In the next step, the 100 μl of substrate solution was added to each well and incubated for 30 min, and then the 100 μl of stop solution was added to each well. In the final step, the optical density is assayed at 540 nm.

*Statistical analysis*

The data statistically analyzed using Graph Pad Prism (version 9) and presented as mean ± SD. We used t-test, one-way and two-way ANOVA tests with Tukey and Bonferroni post hoc tests, respectively. The one-way ANOVA test was used for the determinations of SDH activity, and cytochrome c release. The two-way ANOVA test was used for the determinations of mitochondrial ROS level and collapse of MMP. P < 0.05 defined as significant.

## Results

Effect of *P. melanostigma *Venom on Mitochondrial Succinate Dehydrogenase (SDH) Activity

**Figure 1:**
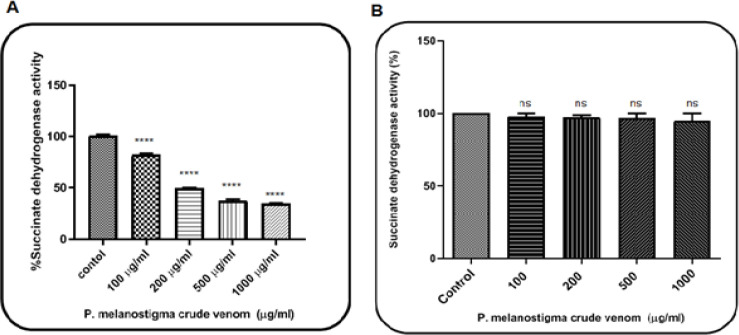
The Effect of *P. melanostigma *Crude Venom on Mitochondrial Succinate Dehydrogenase Activity Measured by MTT Assay Following 60 Minutes of Treatment in the Mitochondria Obtained from U87 Cells (A) and Healthy Group (B). Values are represented as mean± SD (n = 3). ****significant difference in comparison with the corresponding control mitochondria (P < 0.0001)

The different *P. melanostigma *crude venom concentrations (0, 100, 200, 500, and 1,000 µg/ml) inhibitory effect on mitochondrial SDH activity was performed by MTT assay in isolated mitochondria from normal rat brain and U87 cells. Crude venom had a significant (P <0.0001) reduction of mitochondrial SDH activity on glioma mitochondria but not healthy mitochondria (Figure 1A and 1B). 

The IC_50_ value was 230 µg/ml. In the present study, to determine mitochondrial parameters, we used 115, 230, and 460 µg/ml concentrations, in correspondence to IC_50/2_, IC_50_, and 2IC_50_ of this venom.

*Mitochondrial ROS Formation*

**Figure 2:**
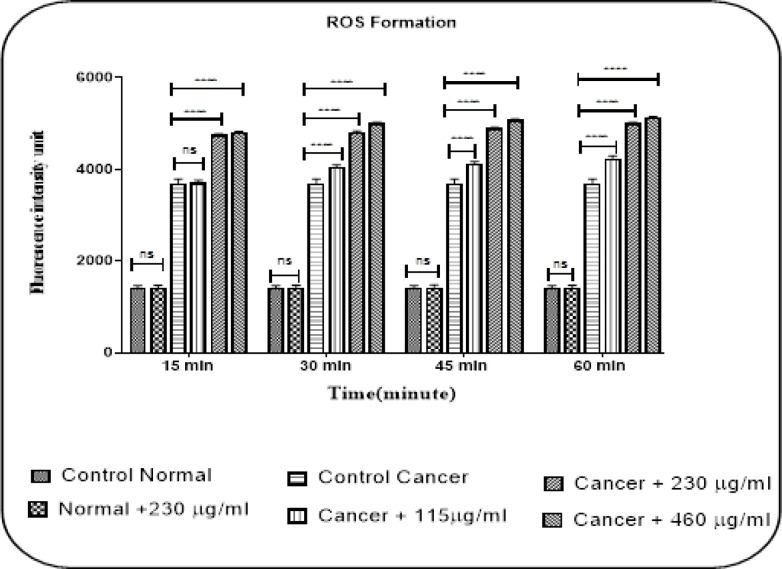
Effects of Stonefish Crude Venom on ROS Formation in the Mitochondria Obtained from U87 Cells and Normal Rat Brain. Stonefish venom induced ROS generation in cancerous groups but not in normal rat brain. Changes mean of fluorescence intensity in ROS generation in mitochondria from U87 cells and normal rat brain treated with Stonefish venom for 60 min summarized in graph. Data presented as mean ± SD. The significant level was p <0.0001 (n =3).

The results showed that stonefish crude venom at concentrations of 115, 230, and 460 µg/ml significantly increase (P < 0.0001) the level of ROS generation in the glioma group at intervals of 15, 30, 45 and 60 min of incubation. On the other hand, this effect has not been observed in isolated mitochondria of the normal group at IC_50_ concentration (230 µg/ml) (Figure 2). 

*Mitochondrial Membrane Potential (MMP) Collapse*

**Figure 3:**
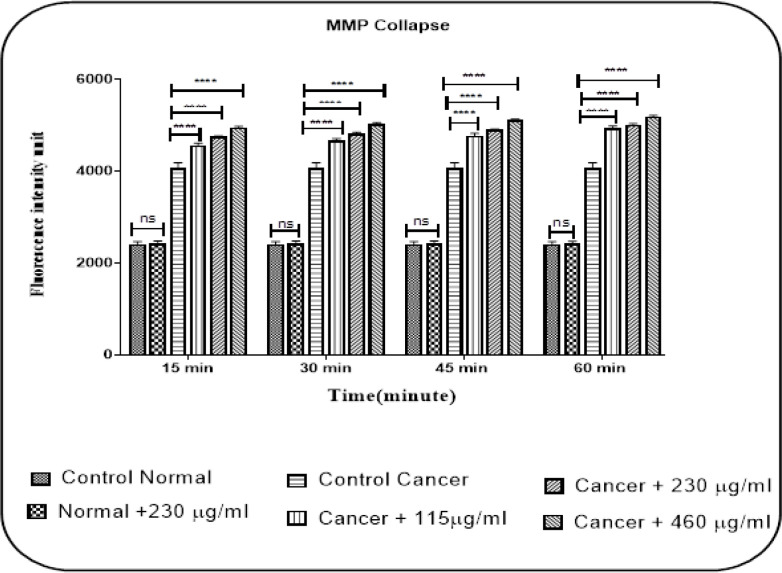
The Effect of Stonefish Crude Venom on MMP Collapse in Mitochondria Isolated from U87 Cells and Normal Rat Brain. Columns show a fluorescence intensity of rhodamine123 in normal and cancerous groups. Data value presented as mean ± SD obtained from three independent assay. ****P<0.0001: significantly different from the corresponding control group

The effect of crude venom on MMP in both glioma and healthy groups has determined by Rh 123 dye. As shown in Figure 3, venom concentrations (115, 230, and 460 µg/ml) have been able to significantly decreased (P < 0.0001) the MMP only in mitochondria from the U87 cells. However, there is no significant collapse in healthy mitochondria following the addition of IC_50_ concentration (230 µg/ml) at intervals of 15, 30, 45 and 60 min of incubation.

*Mitochondrial Swelling*

**Figure 4:**
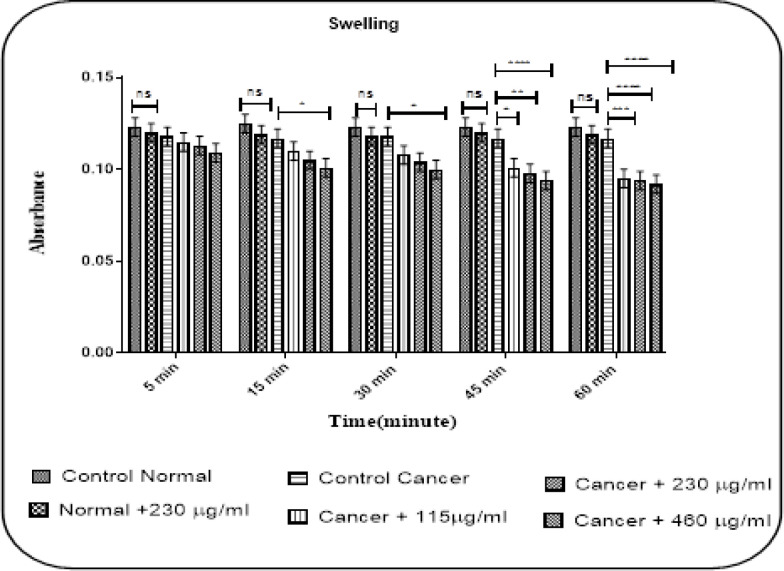
Effect of Various Concentrations of Crude Venom of *P. melanostigma *(115, 230 and 460 µg/mL) on Mitochondrial Swelling at Different Time Intervals within 60 Minutes of Incubation on Isolated Mitochondria from U87 Cells and Normal Rat Brain. Values are represented as mean± SD (n = 3). *P<0.05, **P< 0.01, ***P<0.001, ****P<0.0001

In figure 4 shown, the addition of concentrations of crude venom (115, 230, and 460 µg/ml) led to significant mitochondrial swelling following 45 and 60 minutes of incubation of the glioma mitochondria. Concentration 460 µg/ml also induced significant mitochondrial swelling at 15 and 30 minutes after incubation of the isolated mitochondria from the U87 cells (P< 0.05). Nevertheless, IC_50_ concentration (230 µg/ml) did not induce any mitochondrial swelling in normal mitochondria (Figure 4).

*Cytochrome C Release*

**Figure 5:**
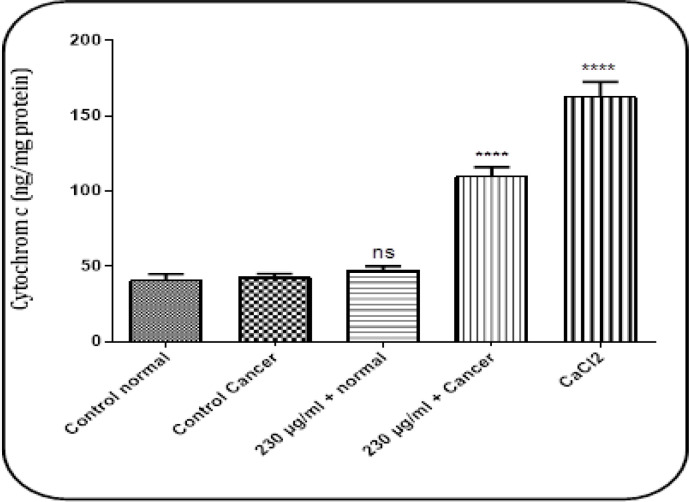
The Effect of Stonefish Crude Venom on Cytochrome c release in Mitochondria Isolated from U87 Cells and Normal Rat Brain. Columns show the amount of cytochrome c release (ng/ mg protein) in normal and cancerous groups. Data value presented as mean ± SD obtained from three independent assay. ****P<0.0001: significantly different from the corresponding control group

When glioma mitochondria were exposed with stonefish crude venom at a concentration of 230 µg/ml (IC_50_) induced significantly (P<0.0001) cytochrome C release. This effect has not been observed in normal mitochondria. Cacl_2_ (100 µM) was used as a positive control for the experiment (Figure 5).

## Discussion

One of the most important non-communicable diseases is cancer, which due to its chronic nature and the burden of the disease, imposes high treatment costs on the health system. Considering the increasing statistics of cancer as well as the high cost of chemotherapy, adverse and toxic reactions of existing drugs, drug resistance and disease recurrence, the importance of research to identify cytotoxic compounds of natural origin without causing toxic effects on non-cancerous cells truly manifests. The goal of this study was to evaluate the toxic effects of *P. melanostigma *venom using mitochondria isolated from U87 cells.

Mitochondria is an organelle that has a role in apoptosis. Lately, Mitochondria have been targeted to cure cancer. The difference between cancerous mitochondria and mitochondria in normal cells exhibits that it is possible to discover agents with a selective effect on cancer cells (Pourahmad et al., 2010). In recent years studies have been reported many compounds induce apoptosis by mitochondrial mediators in cancer cells (Brenner and Grimm, 2006; Biasutto et al., 2010).

The criterion of stonefish venom toxicity is inhibition of mitochondrial succinate dehydrogenase enzyme as an indicator of mitochondrial function and activity and as complex II mitochondrial respiratory chains. Our results show that the studied venom significantly inhibits the activity of mitochondrial succinate dehydrogenase (Figure 1). 

ROS is one of the intracellular molecules that affect various cellular activities including metabolism, differentiation and cell death. Increased continuous production of ROS or disruption of cellular mechanisms ROS removal leads to reach ROS threshold and ultimately cell death. Therefore, by exposing cancer cells to compounds that increase reactive oxygen radicals in the mitochondria and reach the threshold, it may be possible to induce cell death and treat malignancy (Matés and Sánchez-Jiménez, 2000; Pelicano et al., 2003; Pelicano et al., 2004). We show that crude venom significantly (P<0.0001) increased ROS generation in mitochondria obtained from U87 cells but not in healthy cells and this was in line with the above hypothesis. 

A decrease in mitochondrial membrane potential (MMP) is an event that occurs in the mitochondrial pathway of apoptosis and facilitates the exit of apoptotic agents such as cytochrome C from the mitochondria to the cytosol. Then, cytochrome C in the cytosol forms apoptosome with other factors such as apoptotic protease activating factor 1 (Apaf-1) and procaspase-9, and can eventually lead to the final stages of apoptosis (Kowaltowski et al., 2001; Aw, 2010). In our study, we show that crude venom of stonefish could induce MMP collapse and cytochrome c release in U87 cells mitochondria.

Today, the venom of poisonous animals plays an important role in the medical sciences. These toxins can have physiological and pharmacological effects due to their different biologically active molecules. There is a great variety of toxins and their sources that can be considered as a new source for the treatment of diseases (Kemparaju and Girish, 2006).

Previous studies have shown the cytotoxic effect of crude venom and fractions of *P. melanostigma *on ALL lymphocytes and cancerous hepatocytes, as well as its hematologic effects (Aghvami et al., 2017; Mirshamsi et al., 2017a; Babaie et al., 2019). The results of these studies show that crude venom and venom fractions of stonefish can have toxic effects on cancerous lymphocytes and hepatocytes, which has been through mechanisms similar to our study (Aghvami et al., 2017; Mirshamsi et al., 2017a).

In comparison to our study, much research has been done on the effects of the venom of other aquatic and terrestrial animals on different cancer cells. *Naja oxiana *venom had toxicity on cancerous hepatocytes mitochondria (Seydi et al., 2017). *Cassiopea Andromeda *venom fractions induce injury in mitochondria obtained from breast adenocarcinoma patients (Mirshamsi et al., 2017b). *Conus Textile *venom affects glioblastoma through mitochondrial-mediated apoptosis pathway (Salimi et al., 2020). Marine Mollusc (*Turbo Coronatus*) induces apoptosis in mouse melanoma cells (Zangeneh et al., 2018). 

Research on Rhopalurus junceus venom demonstrated the cytotoxic effect of this venom against cancer cell lines of epithelial origin (Dioguardi et al., 2020). Scorpion venom Androctonus Australis has shown that has anti-tumor activity on HCT116, HepG2, MCF-7, and PC-3 as cancer cell lines (Nafie et al., 2020). Lorena Polloni and colleagues reported that *Bothrops Pauloensis* venom has Antiangiogenic effects on endothelial cells (Polloni et al., 2021).

Finally, as a conclusion, this study illustrates that crude venom of stonefish, *Pseudosynanceia melanostigma *by using; a mitochondrial pathway can cause apoptosis in U87 glioblastoma cell mitochondria through mediating increased ROS and with minor effects on healthy cells.

## Author Contribution Statement

Maral Ramezani contributed to this research in carrying out the experiments, analyzing the data and writing the paper. Fatemeh Samiei contributed to this research in carrying out the experiments and analyzing the data. Jalal Pourahmad contributed to this research in formulating the research question(s), designing the study, carrying it out as thesis supervisor, analyzing the data, and writing paper.

## Data Availability

Data is contained within the article or supplementary material.
